# Real-Time Fault Detection and Diagnosis of CaCO_3_ Reactive Crystallization Process by Electrical Resistance Tomography Measurements

**DOI:** 10.3390/s21216958

**Published:** 2021-10-20

**Authors:** Soheil Aghajanian, Guruprasad Rao, Vesa Ruuskanen, Radosław Wajman, Lidia Jackowska-Strumillo, Tuomas Koiranen

**Affiliations:** 1 School of Engineering Science, LUT University, Yliopistonkatu 34, 53850 Lappeenranta, Finland; tuomas.koiranen@lut.fi; 2 Institute of Applied Computer Science, Lodz University of Technology, 90/924 Lodz, Poland; Guruprasad.rao@p.lodz.pl (G.R.); radoslaw.wajman@p.lodz.pl (R.W.); lidia.jackowska-strumillo@p.lodz.pl (L.J.-S.); 3 School of Energy Systems, LUT University, Yliopistonkatu 34, 53850 Lappeenranta, Finland; vesa.ruuskanen@lut.fi

**Keywords:** fault detection, reactive crystallization, electrical resistance tomography, CaCO_3_ precipitation

## Abstract

In the present research work, an electrical resistance tomography (ERT) system is utilized as a means for real-time fault detection and diagnosis (FDD) during a reactive crystallization process. The calcium carbonate crystallization is part of the carbon capture and utilization scheme where process monitoring and malfunction diagnostics strategies are presented. The graphical logic representation of the fault tree analysis methodology is used to develop the system failure states. The measurement consistency due to the use of a single electrode from a set of ERT electrodes for malfunction identification is experimentally and quantitatively investigated based on the sensor sensitivity and standard deviation criteria. Electrical current measurements are employed to develop a LabVIEW-based process automation program by using the process-specific knowledge and historical process data. Averaged electrical current is correlated to the mechanical failure of the stirrer through standard deviation evaluation, and slopes of the measured data are used to monitor the pump and concentrations status. The performance of the implemented methodology for detecting the induced faults and abnormalities is tested at different operating conditions, and a basic signal-based alarming technique is developed.

## 1. Introduction

Crystallization has a significant impact on the final characteristics of particulate systems and plays an essential role in the manufacturing stream of various industrial processes such as agricultural chemicals, cosmetics, pigments, food ingredients, and the highly-regulated active pharmaceutical ingredients (APIs). Real-time anomaly detection and monitoring the functioning stability of the physical–chemical components during crystallization processes are crucial to ensure a reliable operation, minimize performance variations, and, therefore, improve product quality and production volumes.

In particular, there has been recent growth in need of chemical industries for precipitation processes, which lies in the extreme requirement of energy-efficient operation, process intensification, and sustainability [1]. In comparison to cooling and evaporative crystallization, precipitation processes, also known as reactive crystallization, can be implemented at a reduced level of thermal energy [2]. In reactive crystallization, supersaturation generation is done by performing a chemical reaction in the solution to form a solid compound at a concentration that is higher than its solubility in the solution [3]. A major unfavorable aspect of the reactive crystallization processes is the potential to generate a high degree of supersaturation, which increases the possibility of crystal aggregation and agglomeration [4].

Considering the extensive applications of crystallization, a wide range of in situ and online instrumentation and distributed sensing technologies have been developed for process monitoring and control, which noticeably improve the quality of the final crystals. For instance, the following sensor technologies have been extensively used: focused beam reflectance measurement [5], particle vision probe [6,7], turbidity measurement [8], electrical [9], and ultrasound tomography [10].

Crystallization monitoring tools provide abundant information from the system that augments the understanding of the unit operation and enables a framework to identify potential problems and abnormal behaviors. Among such distributed sensing measurement systems, process tomography is an emerging technology that delivers the capability of measuring spatio-temporal field information (e.g., species distribution and phase), visualization through image reconstruction, and cross-sectional images (tomographs) of particular property distribution [11].

Tomographic measurements in chemical engineering processes can generate a large amount of data from process parameters and facilitate the route towards developing: (i) pattern recognition for fault detection via machine learning algorithms and data fusion strategies [12], (ii) data-driven behavior characterization in batch processes, and (iii) human–machine interfaces and volumetric visualization [13]. Even though the adoption of new digital and data-driven technologies has become a common practice in the aerospace and automotive industries, its pragmatic implementation in chemical processing manufacturing has not been broadly observed [14]; hence, process tomography has the potential to initiate the transition from point-based measurements to graphical visualization via big data analytics [15].

Electrical resistance tomography (ERT) is one of the fast-evolving tomographic modalities used to characterize a process through quantifying the electrical field of the medium. The fast growth of ERT is mostly due to the economic aspects, the relative simplicity of implementation, and the potential for sub-millisecond temporal resolution [16]. On the other hand, among the main disadvantages is the data acquisition rate, which affects the image reconstruction time; noise generation is highly correlated to measurement speed, therefore, undesirably impacting the image quality [17]. Furthermore, the inherent nonlinearity of both the conductivity measurement and the inverse algorithms limits the spatial resolution of the electrical tomography technique to approximately 2–5% of the reactor diameter [13,16].

Electrical resistance tomography is a subcategory of electrical impedance tomography in which the real component of the impedance is measured. Most applications of ERT in chemical engineering are dedicated to the extent of agitation performance in stirred tank reactors: liquid/liquid mixing [18], solid/liquid mixing [19], mixing of multiphase non-Newtonian fluids [20], investigating gas hold-up distribution [21,22], and also, in air-lift reactors to measure local gas holdup and visualize gas distribution [22]. Few research works investigated the application of ERT to monitor the progression of precipitation processes through tomographic measurements. These studies investigated the feed location and effects of ionic solution addition using barium sulfate precipitation as a study case [23,24]. In a recent, first-of-its-kind investigation, 2D ERT sensor data were integrated with machine learning to monitor reaction-type crystallization development and to demonstrate the prospect of utilizing ERT as a robust tool for simultaneous pH and conductivity measurement [25].

Considering the nature of the ERT, spatially averaged conductivity values allow the measurement to be obtained across the planar region of interest. ERT has the potential for species transformation measurement and reaction progress monitoring through quantifying the topology-based visualizations of reactive crystallization processes. Successful implementation and interpretation of the physical phenomena improves the understanding of the mixing and feed addition in a stirred tank reactor and facilitates process optimization. Additionally, due to the characteristics of the ionic solutions, rapid changes can be recorded and correlated to specific events during the process, for instance, identifying the feed addition time and location, the start of mixing, etc. To the best of our knowledge, utilizing electrical resistance tomography sensor information as a means for real-time fault detection and diagnosis (FDD) during (reactive) crystallization processes has not been investigated.

Common methodologies for fault detection, malfunction identification, and abnormal events characterization in chemical processes are carried out by failure assessment techniques such as fault tree analysis (FTA), Bayesian network (BN), and principal component analysis (PCA) [26]. Even though BN and PCA are identified as superior techniques in handling complex processes, the conventional fault tree has been extensively applied in process systems and fault diagnosis [27,28]. FTA depends on both probability theory and Boolean algebra and can be conducted qualitatively, quantitatively, or as a combination of both [29]. A frequent assumption in FTA is the independence of events, which is not necessarily valid [30]. Moreover, the standard fault tree approach is not flexible enough for large intercorrelated systems [27].

In this paper, a real-time ERT-based fault detection and diagnosis approach for the reactive crystallization process of CaCO_3_ is investigated. A basic qualitative fault tree analysis was carried out to identify the key events during the process, which includes stirrer states, pump, and feed concentration. The utilized voltage exciting-current measurement ERT system comprises a single plane of 16 stainless steel electrodes around the perimeter of the crystallizer. The measured electrical current of a single electrode was used as input to an in-house developed LabVIEW program where dynamic statistical analysis was carried out for process automation, decision making, and alerting system. The strategy for selecting a single ERT sensor was experimentally and quantitatively investigated based on the relative sensitivity criterion of the individual electrode. Theoretical calculations, experimental repetitions, and process-specific knowledge were used to analyze the statistical patterns in the measured electrical current and to ensure the selection of the suitable sensor (electrode). The investigated crystallization process is part of the carbon capture and utilization scheme where process malfunction and monitoring are presented in this research work.

## 2. Materials and Methods

### 2.1. The Integrated $CO_{2}$ Capture and $CaCO_{3}$ Crystallization Setup

The chemical reaction governing the liquid–liquid reactive crystallization of calcium carbonate is presented in Equation (1):

(1) $${\mathrm{CO}}_{3}^{2-}{}_{\left(\mathrm{aq}\right)}+2{\;\mathrm{Na}}^{+}{}_{\left(\mathrm{aq}\right)}+{\mathrm{CaCl}}_{2}{}_{\left(\mathrm{aq}\right)}\to {\mathrm{CaCO}}_{3}{}_{\left(s\right)}↓+2{\;\mathrm{NaCl}}_{\left(\mathrm{aq}\right)}$$

**Figure 1:**
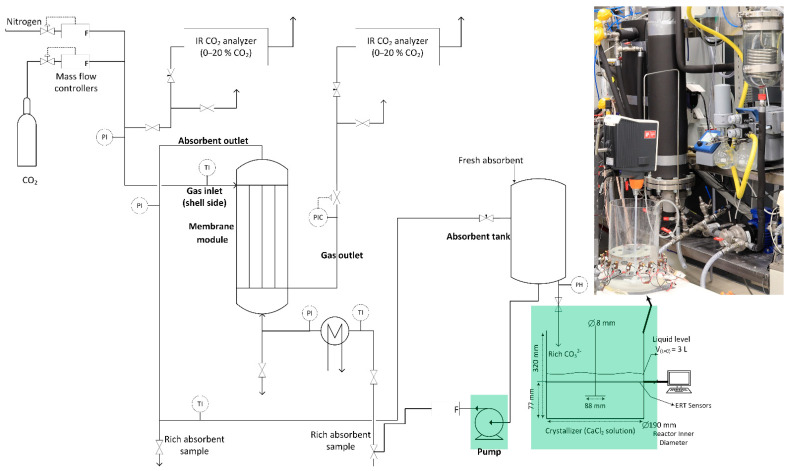
Process schematics: integrated ${\mathrm{CO}}_{2}$ capture and calcium carbonate crystallization. A photograph shows the electrical resistance tomography mounted around the crystallizer. Marked with green shows the main components for the FDD analysis.

In the process under investigation, the semi-batch feed contains dissociated ${\mathrm{CO}}_{3}^{2-}$, ${\mathrm{OH}}^{-}$, and ${\mathrm{Na}}^{+}$ ionic compounds at a pH range of 12.1 ± 0.05. The aqueous ionic solution flows through an inlet pipe (diameter: 3.2 mm) into the receiving reactor containing calcium chloride (${\mathrm{CaCl}}_{2}$, purity > 98%, Merck, Darmstadt, Germany). Figure 1 shows the schematics and a photograph of the entire experimental setup in which the ERT system was utilized for fault detection and diagnosis analysis.

The feed solution is a result of absorbing ${\mathrm{CO}}_{2}$ gas (purity > 99.99%) into a high concentration of sodium hydroxide (NaOH, purity > 98%, Merck) solutions. A magnetic drive gear pump (Pulsafeeder Eclipse E12) was used to circulate the liquid solution in the unit and for the addition of the ${\mathrm{CO}}_{2}$-loaded solution to the crystallizer. The feed addition rate to the receiving reactor is constant at 40 mL $\min^{-1}$ during the entire experimental work.

A gas-liquid hollow fiber membrane contactor (polypropylene, Liqui-Cel 2.5 × 8 Extra-Flow, 3 M) was used for ${\mathrm{CO}}_{2}$ absorption into the NaOH solution. Carbon dioxide absorbs into the liquid solution to form ${\mathrm{CO}}_{3}^{2-}$ through a well-established carbon dioxide instantaneous dissolution and dissociation process in NaOH solutions [30]. Firstly, through the physisorption process, ${\mathrm{CO}}_{2}$ gas is physically absorbed in the liquid phase:

(2) $${\mathrm{CO}}_{2}{}_{\left(g\right)}\to {\mathrm{CO}}_{2}{}_{\left(\mathrm{aq}\right)}$$

Secondly, in the subsequent chemical reactions, which are very fast at higher pH values [31,32], aqueous ${\mathrm{CO}}_{2}$ reacts with hydroxide ions to form ${\mathrm{HCO}}_{3}^{–}{}_{\left(\mathrm{aq}\right)}$ and ${\mathrm{CO}}_{3_{\left(\mathrm{aq}\right)}}^{2-}$, according to Equations (3) and (4), respectively:

(3) $${\mathrm{CO}}_{2}{}_{\left(\mathrm{aq}\right)}+{\;\mathrm{OH}}_{\left(\mathrm{aq}\right)}^{-}\Leftrightarrow {\mathrm{HCO}}_{3_{\left(\mathrm{aq}\right)}}^{-}$$

(4) $${\mathrm{HCO}}_{3_{\left(\mathrm{aq}\right)}}^{-}+{\;\mathrm{OH}}_{\left(\mathrm{aq}\right)}^{-}\Leftrightarrow {\;H}_{2}O_{\left(l\right)\;}+{\mathrm{CO}}_{3_{\left(\mathrm{aq}\right)}}^{2-}$$

Hydrogen carbonate formation occurs immediately after carbon dioxide dissolution (Equation (2)); hence, the aqueous form of the ${\mathrm{CO}}_{2}$ is instantaneously consumed in the solution. Detailed descriptions of the membrane contactor system for the ${\mathrm{CO}}_{2}$ capture unit and its integration with a calcium carbonate crystallization process are given in [33,34], respectively.

### 2.2. The Electrical Resistance Tomography System

**Figure 2:**
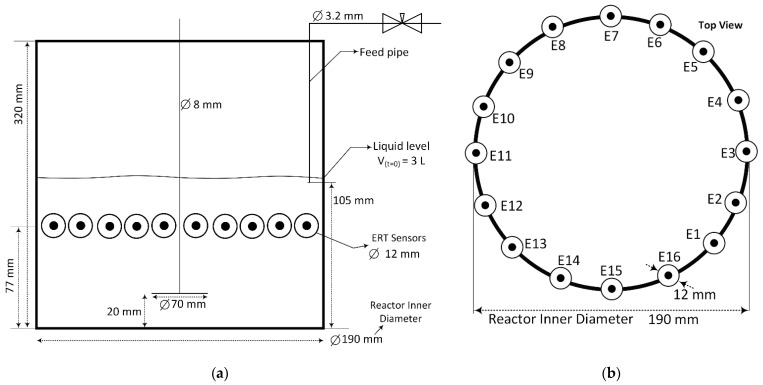
Schematics of the experimental setup. (**a**) Dimensions of the plexiglass reactor and position of the ERT sensors. (**b**) Top view of the array of ERT sensors around the reactor. Electrodes were installed at equal distances around the perimeter of the tank. Feed addition pipe is located between electrodes 3 and 4 throughout the entire experiment. The initial solution volume in the tank is 3 L.

Figure 2 shows the schematics of the experimental reactor and the array of metal electrodes. The crystallization reactor was equipped with a single plane of 16 stainless steel electrodes mounted around the perimeter and connected to a data acquisition system (supplied by Rocsole Ltd., Kuopio, Finland). The utilized electrical resistance tomography was based on the injection of a constant electrical voltage of 2241.34 mV on one of the electrodes (i.e., source electrode) and simultaneously measuring the electrical current distribution on the remaining electrodes (i.e., sink electrodes). The source electrodes were consecutively switched from electrodes number 1–16, and the measurement was recorded accordingly. The frequency of operation is 156 kHz, and the image capturing frame rate is 14.7 Hz.

The circular electrodes with a diameter of 12 mm were made of stainless steel and were configured inside the wall of the reactor. There was no visible oxidation or rust on the electrodes, and they are not interfering with the chemical reaction. Experiments were conducted at a temperature of (20 ± 2) °C. A plastic-made Rushton impeller was used for agitation; in comparison to the metal impeller, the plastic-made impeller reduces the induced noise intensity during the data acquisition process, which is favorable in terms of image reconstruction and statistical signal analysis. The impeller tip speed was kept constant at $0.37{\;m\;s}^{-1}$ for all the experiments.

### 2.3. Failure Identification and Fault Tree Analysis Development

The fault tree analysis methodology was used to develop the system failure states irrespective of their severity. The FTA is a graphical logic representation of combinations of failures or events that may occur to a functional system and shows a map of failure paths. Depending upon the criticality of the process, each branch can be developed further. The logic gates in the FTA are commonly represented by OR and AND gates. In OR gate, system components interactions are in series: failure of any single element leads to failure of the entire process. In AND gate, system components interact in parallel; thus, simultaneous failure of all the system components is required to fail the whole process.

**Figure 3:**
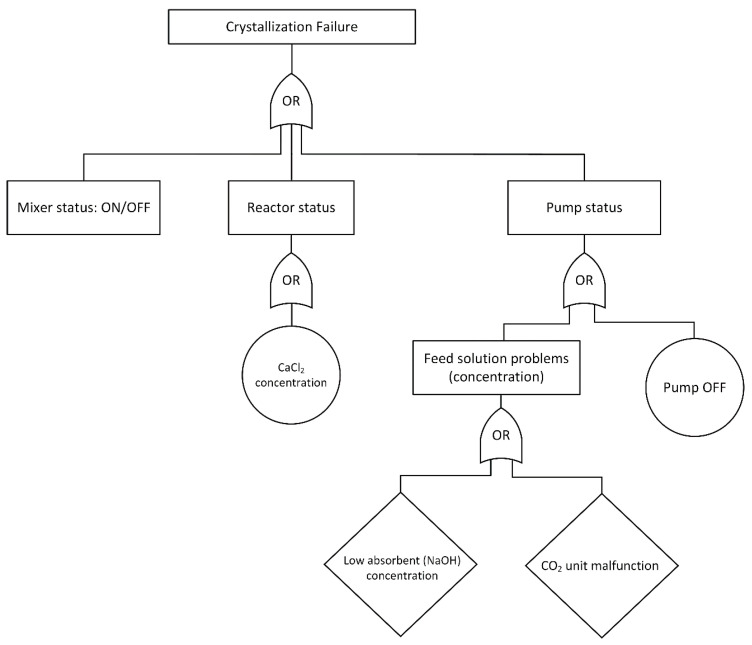
Fault tree analysis diagram for the integrated ${\mathrm{CO}}_{2}$ capture and reactive crystallization process of calcium carbonate.

Figure 3 shows the FTA of the integrated crystallization–${\mathrm{CO}}_{2}$ capture system of the present study. The process under investigation consists of several important physical and chemical components such as feeding pump, stirrer, reagent solution, ${\mathrm{CO}}_{2}$ gas absorbent solution concentration, and amount of calcium chloride in the receiving reactor. Because there is a relatively large theoretical combination of such faults, the investigated cases represent the ones with practical interest to the entire process.

The fault tree comprises a top-level event, intermediate events, and potential root causes for each scenario. Three primary causes induce the failure of the topmost event: stirrer status, initial concentration in the receiving tank (reactor status), or pump status (feed addition). Reagent distribution in the crystallizer is not efficient when the mixer is in switched-off mode and results in extensive aggregation and agglomeration of particles. Moreover, the lack of ${\mathrm{CaCl}}_{2}$ in the reactor, nominal operation of the pump, and concentration of the incoming reagent solution are additional points of failure of the semi-batch crystallization process. Each of the primary events can be a combination of lower-level events; circles represent cases that need no further expansion, and diamonds stand for situations where no additional developments have been investigated (e.g., due to lack of information or scope of the present study).

### 2.4. Malfunction Diagnostics Implementation

Single- and multi-electrode current measurements and real-time statistical analysis of the ERT electrodes can be exploited for real-time fault detection and malfunction diagnosis when out-of-specification events occur throughout the entire process. In the present study, the graphical user interface for data acquisition and malfunction identification was developed in the LabVIEW software environment. A first-in-first-out (FIFO) buffer memory was deployed to store the electrical current measurements from all the electrodes where the newly available measurements continuously substitute the oldest data points in the local history. Moving average and a user-controlled sample length are used for sampling the measurements of a pre-selected electrode. Real-time tuning of the sampling length and intervals provides a rational approximation of the events from inside the suspension and the precipitation process.

Averaged single-electrode data from the ERT system were correlated to the mechanical failure of the stirrer through standard deviation (SD) evaluation. The measured electrical signals were averaged every 5 s, and then, a moving average with a time difference of 20 s was used for dynamic evaluation of the standard deviation, which provides satisfactory performance for basic applications. According to the relation in Equation (5), when the real-time calculation of the SD ratio criterion is greater than a pre-determined parameter, it is an indication of an abrupt change in the status of the mixer (e.g., mixer switched off). The mixer standard deviation ratio criterion ($\sigma_{r}$) was investigated for the scenarios where the feed addition (pump) is on and off.

(5) $$\begin{array}{cc} \begin{array}{c} \sigma_{r}={\frac{\sigma_{\mathrm{mixer}\;\mathrm{off}}}{\sigma_{\mathrm{mixer}\;\mathrm{on}}}|}_{\left(\mathrm{pump}\;\mathrm{on}\right)}\\ \sigma_{r}={\frac{\sigma_{\mathrm{mixer}\;\mathrm{off}}}{\sigma_{\mathrm{mixer}\;\mathrm{on}}}|}_{\left(\mathrm{pump}\;\mathrm{off}\right)} \end{array} & \mathrm{where},\{\begin{array}{c} \mathrm{Mixer}\;\mathrm{on},\;\;\sigma_{r}<\varepsilon_{\mathrm{mixer}}\\ \mathrm{Mixer}\;\mathrm{off},\;\;\sigma_{r}\geq \varepsilon_{\mathrm{mixer}} \end{array} \end{array}$$

where $\sigma_{\mathrm{mixer}\;\mathrm{off}}$ and $\sigma_{\mathrm{mixer}\;\mathrm{on}}$ are the signal standard deviation obtained from experimental data when the mixer is switched off and switched on, respectively. $\varepsilon_{\mathrm{mixer}}$ denotes the trigger value that initiates the alert as the criteria are fulfilled, which is the same for both the pump-on and pump-off situations. The adjustable parameters (i.e.,$\;\sigma_{r}$, sampling times, etc.) are manually fine-tuned for each operating condition by trial-and-error procedures according to the stability and transient response of the experiment under investigation.

Similarly, ascending and descending slope of the averaged electrical current from the designated electrode were used to monitor the pump and feed addition status. The formation of the solid particles in the solution decreases the mean electrical current of the solution throughout the feed addition window. The average slope of the electrical current is continuously determined as follows:

(6) $$C=\frac{I}{\Delta t}$$

where I is the electrical current (microampere, μA), C is the average slope of the electrical current (${\mu A\;\min}^{-1}$), and Δt is the measurement time (minute). An abrupt decrease in the continuous slope measurement toward a plateau (i.e., zero slopes) indicates a situation where the feed addition pump is switched off, as expressed in Equation (7):

(7) $$K=\frac{C_{\mathrm{pump}\;\mathrm{on}}}{C_{\mathrm{pump}\;\mathrm{off}}}\;\mathrm{where},\{\begin{array}{r} \mathrm{Pump}\;\mathrm{on}\;\left(\mathrm{feed}\;\mathrm{addition}\right),\;\;K<\varepsilon_{\mathrm{feed}}\\ \mathrm{Pump}\;\mathrm{off}\;\left(\mathrm{no}\;\mathrm{feed}\;\mathrm{addition}\right),\;\;K\geq \varepsilon_{\mathrm{feed}} \end{array}$$

where K is the slope factor, and $\varepsilon_{\mathrm{feed}}$ is the threshold value that triggers the alert concerning the pump status. A time difference of 30 s was used to evaluate the moving average of the incoming electrical signals for the pump status. Table 1 lists the threshold values for $\varepsilon_{\mathrm{mixer}}$ and $\varepsilon_{\mathrm{feed}}$ that were used during the experimental investigation.

### 2.5. Total Ion Balance Modeling and the Minimum Runtime

The presence of different ionic compounds (see Equation (1)) during the calcium carbonate crystallization process results in the variation of the electrical current flow in the opaque solution. To enhance the accuracy of the process automation in terminating the crystallization (i.e., switching off the mixer and feed pump), a minimum runtime criterion was defined. The theoretical minimum runtime was determined by the total ion balance modeling of the entire process—that is, the minimum amount of the feed solution required to consume a known calcium chloride concentration in the receiving tank. A final command to terminate the crystallization process is applied when both the experimentally measured slope factor criterion is satisfied, and the minimum runtime has elapsed.

The data presented in Table 2 were used to perform the theoretical ion balance modeling over time for the investigated chemical reaction. The pumping capacity of the impeller, Q, can be used to quantify the flow pattern in the stirred tank reactor. As expressed in Equation (8), Q is the volumetric flow rate passing through the mixing planes due to the rotation of the impeller [35].

(8) $$Q=N_{q}{\mathrm{ND}}^{3}$$

where $N_{q}$ is the pumping number of the Rushton impeller [36], N is the stirring rate (rps), and D is the impeller diameter.

**Figure 4:**
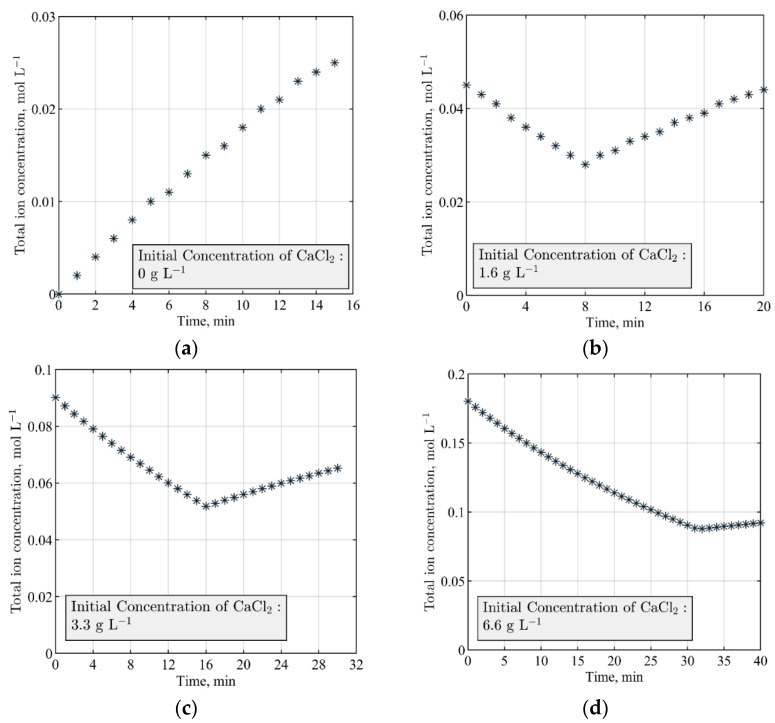
Theoretical modeling and calculation of the total ion balance during the crystallization process. Constant parameters are feed solution pH: 12.1, ${\mathrm{CO}}_{3}^{2-}$ concentration: 0.14 ${\mathrm{mol}\;L}^{-1}$, feed addition rate: $40{\;\mathrm{mL}\;\min}^{-1}$. Initial solution volume in the reactor is 3 L. (**a**) No precipitation; (**b**) precipitation until 8 min, afterward calcium chloride is depleted; (**c**) precipitation until 16 min, afterward no calcium chloride is present; (**d**) precipitation until 31 min, afterward no calcium chloride is remaining.

As presented in Figure 4, four different initial concentrations of calcium chloride were employed to conduct the calculations. When the initial concentration of ${\mathrm{Ca}}_{\left(\mathrm{aq}\right)}^{2+}$ in the receiving reactor is 0 $g\;L^{-1}$, the addition of the ionic feed solution results in an immediate rise in the total ions in the crystallizer without the formation of any solid particles. Crystallization occurs when the initial concentration of ${\mathrm{Ca}}_{\left(\mathrm{aq}\right)}^{2+}$ is non-zero, which is an indication of a decrease in the supersaturation and formation of non-conductive solid particles. Depletion of the initial ${\mathrm{Ca}}_{\left(\mathrm{aq}\right)}^{2+}$ in the reactor results in a continuous descending trend for the global electrical current distribution of the solution.

## 3. Results and Discussion

### 3.1. ERT Sensitivity Analysis and Sensor Selection

**Figure 5:**
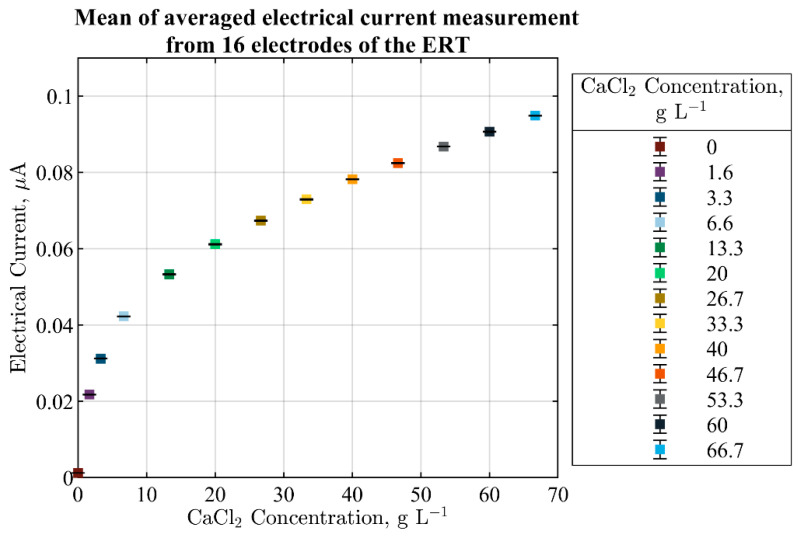
Mean of averaged current measurement from 16 electrodes of the ERT for quantification of the total current of the solution. Different concentrations of ${\mathrm{CaCl}}_{2}$ solutions are used for a 1-min measurement. Solutions are stable with no mixing.

The operating architecture of the utilized ERT system is voltage exciting and current measurement via a system of equidistant electrodes fixed around the boundary of the stirred tank reactor. Gauss–Newton reconstruction algorithm was used for post-processing the ERT tomographs. Even though tomographic data can provide cross-sectional representation from within a stirred tank reactor, in the current investigation, the ERT reconstructions could not be utilized due to non-deterministic and insensitive data from the crystal suspensions. Employing them for fault identification does not efficiently characterize malfunction situations. Moreover, the complex nature of the topology-based visualization renders the real-time interpretation a challenging task. The transient correlation of the reconstructions to physico-chemical phenomena of the fast-kinetic crystallization process becomes extremely limited due to a relatively slow reconstruction time and the global resolution. In summary, more research efforts are needed for image reconstructions studies in detail. On the other hand, statistical analysis of the single- and multi-electrode current measurement of the ERT provides satisfactory information from the suspension that contains a conductive liquid phase and non-conductive solid phase. Figure 5 displays the multi-electrode current measurements for different concentrations of water-${\mathrm{CaCl}}_{2}$ system. The mean of averaged electrical current from 16 electrodes is used for approximate quantification of the total current of the solution. Results show that the distinguishability and sensitivity range of the utilized ERT system becomes limited by operating at a relatively higher ${\mathrm{CaCl}}_{2}$ concentration.

**Figure 6:**
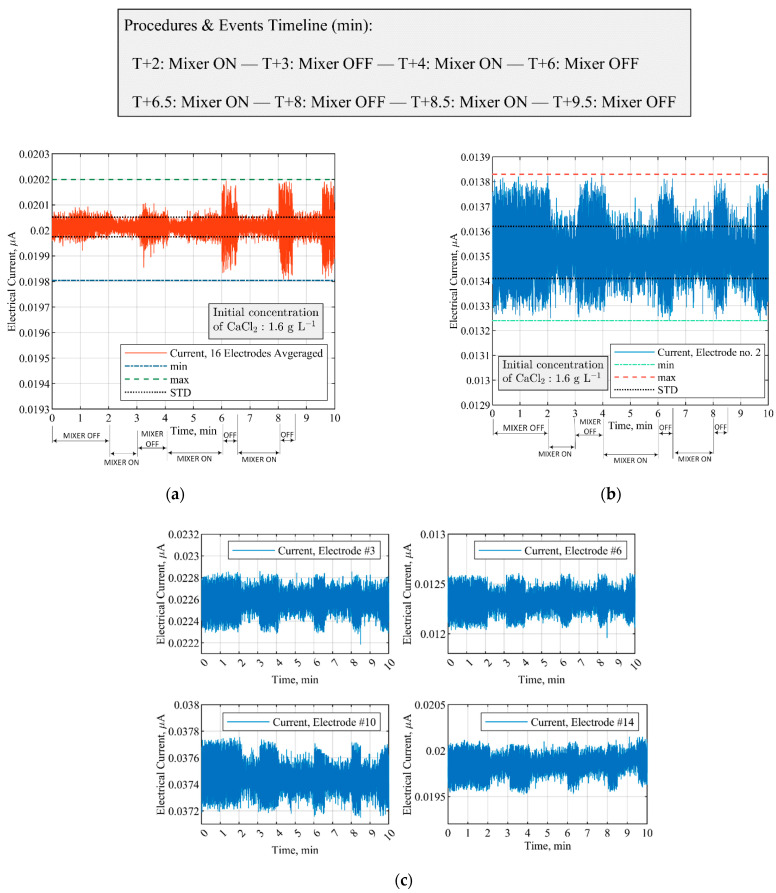
Changes in electrical current measurement induced by switching on (100 RPM) and off (0 RPM) the stirrer at ${\mathrm{CaCl}}_{2}$ concentration of 1.6 $g\;L^{-1}$, without any reagent addition. (**a**) Electrical current measurements by averaging 16 electrodes; (**b**) mixer-induced variation in the signals acquired through electrode no. 2; (**c**) comparison of electrical current measurements via single-electrode located at 90-deg apart inside the stirred tank reactor. Quantitative information from all the electrodes is reported in Appendix A.

Additional statistical analysis of the electrical currents from ERT electrodes demonstrates a continuous pattern in the acquired signals that can be linked to physio-chemical changes of the precipitation process. As discussed in Section 2.4, the effects of mixer status (i.e., switched on/off) can be observed through standard deviation analysis. Figure 6 shows the variation in electrical current measurement based on a single- and multi-electrode analysis induced by the change in stirrer conditions. The elapsed time (T) of the experiment is reported in the form of T+ (T-Plus), which starts from the beginning of the process. Upon switching off the mixer at times 3, 6, 8, and 9.5 min into the process, an abrupt increase in the relative standard deviation of the measurement data is recorded. According to Table 1 (Section 2.4), a threshold value of 1.9 ± 0.1 was assigned to the mixer SD ratio ($\sigma_{r}$) for monitoring and reporting the stirrer operating status. The assigned value to $\sigma_{r}$ was obtained through experimental repetitions and trial-and-error procedures. If the dynamic SD ratio measurements rise above the threshold value, it triggers an alert indicating the mixer is switched off.

The rise in signal amplitude due to a change in the mixer status is robustly observed over electrode no. 2 (Figure 6b)—a quantitative analysis of the standard deviation ratio of all the electrodes of the utilized ERT system for the selected batch experiment is presented in Appendix A (Table A1). Calculating the SD ratio around the time that the mixer is switched off indicates the minimum SD ratio criterion is satisfied over electrode no. 2. Even though analogous patterns are spotted in other electrodes—including the total averaged value (Figure 6a)—the evaluated $\sigma_{r}$ is consistent on the selected electrode throughout different experimental repetitions.

Although employing a single electrode to analyze the ERT measurements provides partial knowledge of the suspension and the precipitation process, it demonstrates as a promising tool to investigate faults and malfunction throughout the process. In the present study, the measurement of the electrical current from electrode no. 2 was used to develop the FDD methodology. The selected electrode was located close to the feed point and demonstrated a relatively higher sensitivity to the process under investigation.

**Figure 7:**
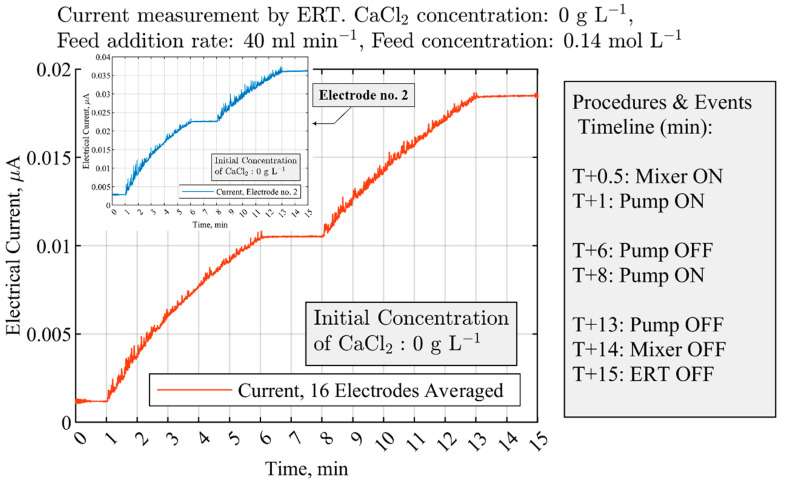
Experimental measurements of the electrical current with the ERT system; averaged current from 16 electrodes is compared the measurements of the electrode no. 2. The mixer continuously works at 100 RPM.

A good qualitative agreement was identified between the theoretical analysis of the total ion balance (Figure 4a) and the experimental measurements by the ERT electrodes. Figure 7 shows the measurements where the initial amount of ${\mathrm{Ca}}_{\left(\mathrm{aq}\right)}^{2+}$ is 0 $g\;L^{-1}$; hence, the addition of the ionic solution (containing ${\mathrm{OH}}^{-}$ and ${\mathrm{CO}}_{3}^{2-}$) to water results in a continuous increase in the overall electrical current. While the mixing is uninterrupted at 100 RPM during the experiment, the pump switches off at 6 min into the process for monitoring the effects of the feed addition; after switching on the pump at T+8 min, a similar pattern is observed. Measurements were obtained by averaging the electrical currents from 16 electrodes of the ERT system and compared with the data from electrode no. 2. Additional qualitative similarities between the theoretical analysis and the experimental measurements are discussed in Section 3.3.

### 3.2. Measurements of CaCO_3_ Solid Particles Addition by ERT Electrodes

To investigate the dynamical effects of solid calcium carbonate on the electrical field of the ERT system, two different amounts of solid particles were added into the reactor containing 3 L water. Solid samples of commercial calcite (provided by VWR, purity > 99%) were used for the measurement. Particles were added from the top of the reactor by hand.

**Figure 8:**
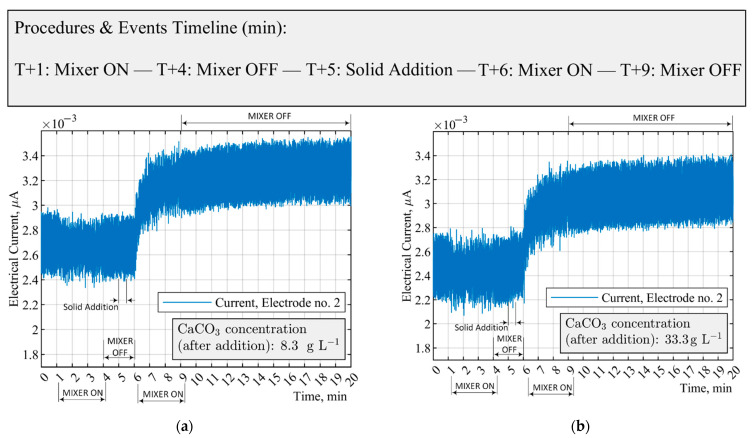
Variation in the electrical current of CaCO_3_ suspensions based on single-electrode measurements; (**a**) CaCO_3_: 8.3 ${g\;L}^{-1}$; (**b**) CaCO_3_: 33.3 ${g\;L}^{-1}$. The mixer switches on at 100 RPM.

Figure 8a,b shows the experimental measurement and procedures for the solid concentrations of 8.3 $g\;L^{-1}$ and 33.3 $g\;L^{-1}$. Solid particle addition occurred after 5 min (i.e., T + 5 min) into the measurement when the mixer was switched off from 100 RPM. No significant changes in the electrical current of the medium were observed before and after the addition of the non-conductive calcium carbonate. The electrical current of the solution slightly increases from an average value of $\left(2.7\pm 0.3\times 10^{-3}\right)$ µA to $\left(3.3\pm 0.2\times 10^{-3}\right)$ µA after switching on the mixer at T+6 min. The relative increase in the current is primarily due to the distribution of solids and distinct electrical properties of the suspension. Moreover, since the background medium was non-conductive in comparison to ${\mathrm{Ca}}_{\left(\mathrm{aq}\right)}^{2+}$ solutions, the status of the mixer (e.g., on/off) was not substantially affecting the electrical field.

### 3.3. ERT-Based Fault Detection and Malfunction Scenarios

Different scenarios of the ERT-based malfunction investigation are listed in Table 3. Existing patterns in the signals measured by a single electrode of the ERT system are analyzed to determine the mechanical equipment condition throughout each batch operation.

In all the cases, the initial concentration of calcium chloride was 1.6 $g\;L^{-1}$. The main operating parameters of the experiments are tabulated in Table 2 (Section 2.5). The end-to-end experimental procedure for each operating condition was repeated three times to ensure the validity and repeatability of the results. As presented in the total ion balance modeling of the process, the addition of the ionic reagent solution to the reactor leads to the precipitation of ${\mathrm{CaCO}}_{3}$ and the subsequent depletion of ${\mathrm{CaCl}}_{2}$. The formation of the non-conductive solid calcium carbonate was monitored in real-time by the ERT system and measured through electrode no. 2, which was located adjacent to the feeding point.

**Figure 9:**
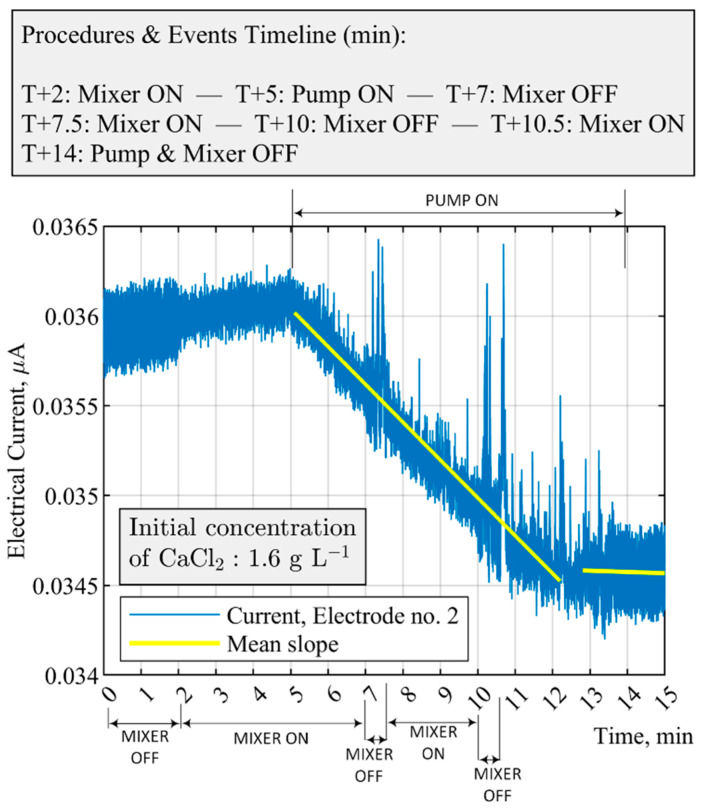
Fault detection and malfunction scenarios for case no. 1 in Table 3. The feed pump is constantly switched on, and the mixer switches on (100 RPM) and off (0 RPM), while the precipitation is ongoing. The entire experimental process automatically shuts down when ${\mathrm{CaCl}}_{2}$ is depleted at ca. T + 13 min. The constant feed flow rate is 40 mL $\min^{-1}$.

Figure 9 displays a situation where operator-induced mixer malfunctions were enforced while the precipitation process was ongoing; mixer malfunctions of duration 30 s were applied at times T + 7 min and T + 10 min into the process. Switching off the stirrer results in a rapid variation of the electrical current and increases the mean standard deviation, which in turn notifies the operator via an alert system implemented in the process automation program. Currently, return to the nominal operating mode following the fault is done by the operator; however, there is a possibility of full automation by further development of the experimental setup and the software.

Furthermore, as shown in Figure 9**,** the slope of the measured electrical current has a descending trend because of the continuous reagent addition and formation of solids. The decreasing slope of the fitted linear line between 5–12 min, which equals ca. $-2.12\times 10^{-4}$ ${\mu A\;\min}^{-1}$, indicates the consumption of ${\mathrm{CaCl}}_{2}$ in the suspension. After 13 min into the process, the value of the slope decreases ($-5.68\times 10^{-6}$ ${\mu A\;\min}^{-1}$) to a roughly plateau region. For the employed concentrations of species, both an abrupt change in the slope factor after 13 min and fulfilling the minimum experimental runtime result in an automatic shutdown of the mixer and the feed pump. Quantitative data of the mean value of the slope between 5 and 12 min from different electrodes are presented in Table 4. The data address the reliability of the selected electrode in triggering the alert system concerning the stirrer malfunctions; in comparison to other electrodes, steep of the slope in electrode no. 2 is greater.

**Figure 10:**
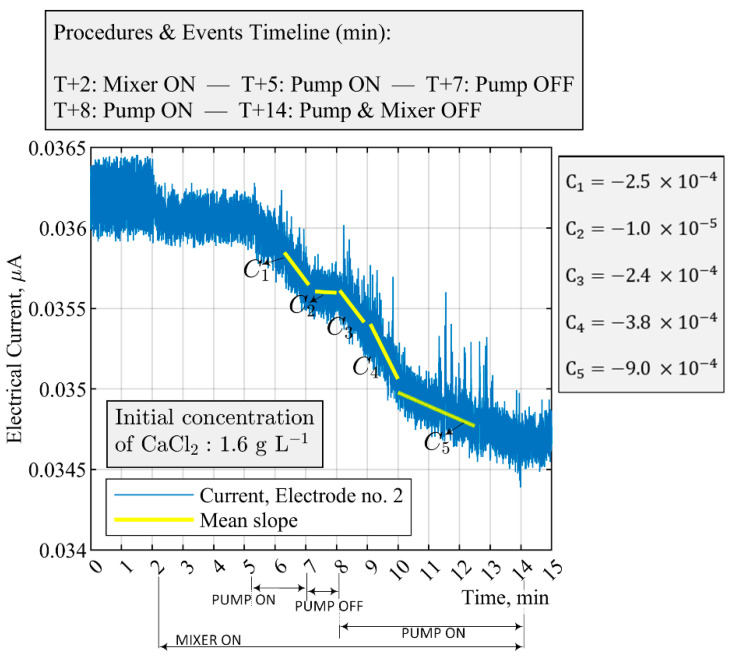
Fault detection and malfunction situation for case no. 2 in Table 3. The stirrer is continuously on (100 RPM), and a malfunction is enforced on the pump at T + 7 min. Precipitation is ongoing only when the feed pump is on. The feed flow rate is 40 mL $\min^{-1}$.

Figure 10 demonstrates the intermittent operation of the feed pump and its effect on the electrical current measurement of the ERT system (electrode no. 2). The mean value of the electrical current from T + 5 min to T + 7 min has a steeper slope angle in comparison to the period in which the pump is switched off (between 7–8 min). A rapid decrease of 20–25 times in the average slope factor of the electrical current toward a plateau automatically triggers the alarm that indicates a malfunction in the pump—a time difference of 30 s was used to evaluate the moving average of the incoming electrical signals for the pump status. Since the precipitation and the real-time fault detection were dynamic and fast, the accuracy and efficiency of the decision-making of the process automation are promising enough to realize the overall trend of the experimental system.

**Figure 11:**
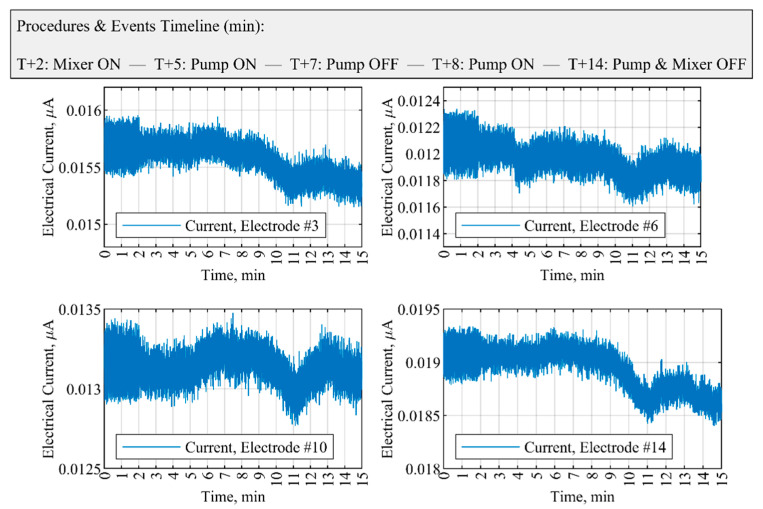
Performance of different sensors during feed pump failure situations (case no. 2 in Table 3). Sensors are separated at 90-deg apart inside the crystallizer. The initial ${\mathrm{CaCl}}_{2}$ concentration is 1.6 $g\;L^{-1}$. The feed flow rate is 40 mL $\min^{-1}$, and mixing speed is 100 RPM.

To ensure a satisfactory single-sensor operation during the precipitation process, the performance and sensitivity of different electrodes of the ERT system to pump failure were analyzed. As displayed in Figure 11, measurements obtained from four equidistance electrodes, positioned at 90-deg apart inside the reactor—namely, sensors no. 3, 6, 10, and 14—can be qualitatively compared with the data from electrode no. 2. In terms of the lower stochastic behavior of the acquired signals in different operating conditions, measurements obtained through sensor no. 2 were, in comparison, robust enough for malfunction identification for the pump.

In addition, Table 5 presents the slope factor evaluation for all the electrodes of the ERT system at critical times during the process—the analysis is used to assess the sensitivity of the measurement point. The implemented methodology based on electrode no. 2 did well in reacting to significant variations in the slope factor, which is an indication of the pump malfunction.

**Figure 12:**
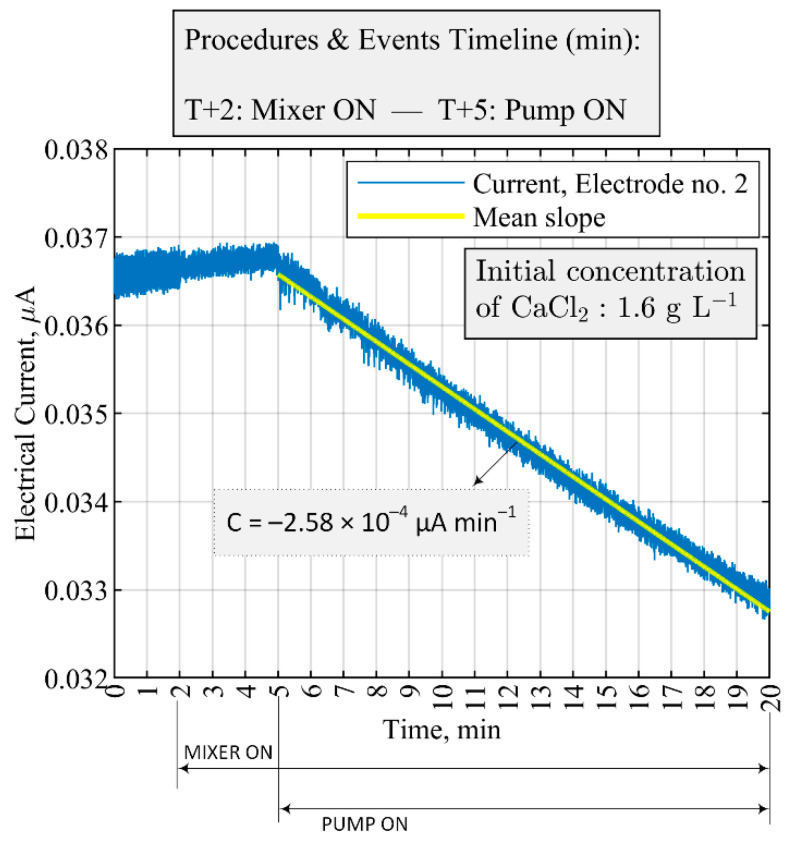
Fault detection and malfunction scenario by a single sensor of the ERT system corresponding to case no. 3 in Table 3. A normal operation for the mixer (100 RPM) and pump ($40{\;\mathrm{mL}\;\min}^{-1}$ ) but the feed solution that flows into the crystallizer contains 0 ${g\;L}^{-1}$ ${\mathrm{CO}}_{3_{\left(\mathrm{aq}\right)}}^{2-}$.

According to the FTA analysis, the content of the feed solution can lead to reactive crystallization failure. Issues in the feed solution could be due to prior faults in the system, for instance, inefficient ${\mathrm{CO}}_{2}$ capture process leads to lower concentrations of the ${\mathrm{CO}}_{3_{\left(\mathrm{aq}\right)}}^{2-}$ in the feed. Figure 12 presents an operating condition where the feed concentration was not at the nominal level of 0.14 $\mathrm{mol}\;L^{-1}$ ± 0.2 $\mathrm{mol}\;L^{-1}$ of ${\mathrm{CO}}_{3_{\left(\mathrm{aq}\right)}}^{2-}$, and instead, water was used to conduct the experiments. The addition of the non-ionic solution to the crystallizer containing 1.6 ${g\;L}^{-1}$ of ${\mathrm{CaCl}}_{2}$ resulted in a continuous decrease in the overall electrical current of the medium, as measured by electrode no. 2. The expected precipitation time after the start of the feed pump was 8 min; since no calcium chloride is consumed throughout the 8-min window, the decrease in the overall electrical current was due to an increase in the solution volume and not the precipitation process.

## 4. Conclusions

In this paper, real-time malfunction diagnosis and fault detection in the reactive crystallization process of ${\mathrm{CaCO}}_{3}$ was investigated by utilizing a single sensor of an electrical resistance tomography system. The occurrence of unfavorable faults and malfunctions in the key components of the process stream negatively impacts the final product and results in crystallization failure and can be quantitatively studied.

The fault detection and diagnostics of the investigated process were developed based on the fault tree analysis approach. It has been shown that failures in the critical process parameters such as mixing, pump, and concentrations can be effectively identified by the existing patterns of a single-electrode measurement. Stirrer operational condition was developed based on standard deviation analysis of the incoming signals, while the electrical current slope assessment was used for reporting the pump (feed addition) situation. To ensure a reliable single-electrode operation, experimental repetitions and quantitative analysis of the standard deviation and slope factors are thoroughly investigated. The process automation program was developed in the LabVIEW software environment, and the threshold values to trigger the alert system were experimentally investigated.

Although the tomograph reconstructions failed for the reasons mentioned in this paper, it was possible to develop a fully functional fault detection system using ERT sensor data. The most sensitive measurement point based on the experimental measurements was selected as sensor data transmitter to fault diagnosis.

The current fault detection approach may be studied by varying the input parameter values broadly to check system sensitivity and robustness. In this context, investigation of different signal processing schemes (e.g., more comprehensive signal-to-noise ratio analysis) could be tested in malfunction identification. Adding more automation to the associated program, such as the capabilities to dynamically identify the most sensitive electrode, can be the subject of further investigations. Additionally, a key area of further research concerns the development of ERT reconstructions and investigates its potential to be used in the process under investigation. Subsequently, the integrated approach (i.e., fault detection and tomography) provides a means to simultaneously study the particle suspension densities at the outlet section and inside the crystallization reactor.

## Figures and Tables

**Table 1:** Threshold values to trigger the alert system for mixer and feed addition pump malfunctions. Averaged values obtained from multiple experiments with their standard deviation are reported.

Equipment	Parameter	Threshold Value	The Time Difference for Moving Average, s
Mixer	$\varepsilon_{\mathrm{mixer}}$	1.9 ± 0.1	20
Pump	$\varepsilon_{\mathrm{feed}}$	15 ± 4	30

**Table 2:** Main operating parameters for the theoretical ion balance modeling and precipitation experiments.

Parameter	Unit	Value
${\mathrm{CaCl}}_{2}$concentrations	${g\;L}^{-1}$	0, 1.6, 3.3, and 6.6
NaOH concentration at the feed	${\mathrm{mol}\;L}^{-1}$	12.1 ± 0.05
${\mathrm{CO}}_{3_{\left(\mathrm{aq}\right)}}^{2-}$concentration at the feed	${\mathrm{mol}\;L}^{-1}$	0.14 ± 0.2
Impeller pumping capacity	$m^{3}{\;s}^{-1}$	0.004
Impeller pumping number	–	0.70
Impeller diameter	m	0.07
Stirring rate	rps	1.67
Impeller tip speed	${m\;s}^{-1}$	0.37

**Table 3:** The investigated scenarios for fault detection and diagnostics with electrode no. 2 of the ERT system. All the experiments are carried out at a mixing speed of 100 RPM and feed addition rate of $40{\;\mathrm{mL}\;\min}^{-1}$ .

Experiment Index	Constantly Operational	Malfunction(s) during the Process
Case no. 1	Pump ON	Mixer ON and OFF
Case no. 2	Mixer ON	Pump ON and OFF
Case no. 3	Pump ON and Mixer ON	Feed is water (not carbonate ions)

**Table 4:** Assessment of the slope of the electrical current between 5 and 12 min from all the electrodes of the ERT system. The data describe the malfunction scenarios for case no. 1 in Table 3 and Figure 9.

The Slope of the Electrical Current from All the Electrodes of the ERT System during the Time between 5 and 12 Min																
Electrode	1	2	3	4	5	6	7	8	9	10	11	12	13	14	15	16
Current (${\mu A\;\min}^{-1}$), × ($-1\times 10^{-4}$)	1.93	2.12	1.04	0.47	0.96	0.14	0.18	0.26	0.19	0.14	0.03	0.11	0.21	0.79	1.51	1.54

**Table 5:** Comparison of the slope of electrical current over all the electrodes of the ERT system. The slope factor, K , is calculated at the time that the pump is switched off, which triggers an alert based on electrode no. 2. The data illustrate the malfunction scenarios for case no. 2 in Table 3 and Figure 10 and Figure 11.

	$t_{1}=6.3–7.1\;\mathrm{Min},$ (Pump On)	$t_{2}=7.2–8\;\mathrm{Min},$ (Pump Off)	Slope Factor, K	$t_{3}=8.1–8.9\;\mathrm{Min},$ (Pump On)
Electrode Index	$C_{t_{1}}$ $\left(\times 10^{-5}\right)$	$C_{t_{2}}$ $\left(\times 10^{-5}\right)$	$K=C_{t_{1}}/C_{t_{2}}$	$C_{t_{3}}$ $\left(\times 10^{-5}\right)$
1	−23.38	−3.16	7.39	−22.98
2	−25.35	−1.01	25.09	−24.87
3	−11.00	−4.71	2.33	−3.15
4	−6.19	−2.98	2.07	−9.55
5	19.12	−10.14	−1.88	−5.62
6	−0.50	−4.04	0.12	0.50
7	1.43	−3.4	−0.42	0.659
8	1.38	−5.46	−0.25	−1.06
9	2.35	−4.6	−0.51	−1.61
10	1.40	−3.10	−0.45	−2.20
11	3.08	−2.10	−1.4	0.78
12	1.86	−2.76	−0.67	−0.48
13	0.16	−1.57	−0.03	−0.18
14	−2.50	−5.00	0.5	−5.61
15	−14.04	−4.58	3.06	−13.39
16	−19.9	−2.36	8.43	−21.29

## Data Availability

The data presented in this study are available on request from the corresponding author.

## Appendix A

**Table A1:** Standard deviation ratio analysis of all the electrodes of the ERT system. SD ratio $\left(\sigma_{r}\right)$ are evaluated according to Equation (5) in the main text and are reported around the time that the mixer switches off. The results are related to the experiment where ${\mathrm{CaCl}}_{2}$ concentration is 1.6 $g\;L^{-1}$, without the feed solution addition; the mixer switches on at 100 RPM. Illustrations for selected electrodes are displayed in Figure 6 of the main text.

	3–3.5 Min, (Mixer Off)	5.5–6 Min	6–6.5 Min, (Mixer Off)	7.5–8 Min	8–8.5 Min, (Mixer Off)
Electrode Index	$\sigma_{r}$	$\sigma_{r}$	$\sigma_{r}$	$\sigma_{r}$	$\sigma_{r}$
1	1.86	0.71	1.53	0.59	1.62
2	2.14	0.55	2.30	0.42	2.12
3	1.40	0.69	1.44	0.68	1.86
4	1.57	0.57	1.60	0.67	1.36
5	1.28	0.97	1.40	0.48	2.01
6	1.84	0.59	1.45	0.65	1.48
7	1.51	0.57	1.04	1.99	1.00
8	1.80	0.55	1.59	0.89	1.08
9	0.56	2.26	1.61	0.75	1.32
10	1.62	0.52	1.58	0.68	1.87
11	1.39	0.64	1.25	0.67	1.86
12	1.66	0.66	1.56	0.61	1.73
13	1.55	0.79	1.61	0.64	1.56
14	1.13	0.84	1.43	0.55	1.56
15	1.52	0.73	1.54	0.73	1.45
16	1.60	0.61	1.79	0.54	1.58
